# Interstage mortality for functional single ventricle with heterotaxy syndrome: a retrospective study of the clinical experience of a single tertiary center

**DOI:** 10.1186/1749-8090-8-93

**Published:** 2013-04-16

**Authors:** Jinyoung Song, I-Seok Kang, June Huh, Ok Jeong Lee, Geena Kim, Tae Gook Jun, Ji Hyuk Yang

**Affiliations:** 1 Department of Pediatrics, Samsung Medical Center, Sungkyunkwan University School of Medicine, 50 Irwon-dong, Gangnam-gu, Seoul 135-710, South Korea; 2 Department of Thoracic and Cardiovascular Surgery, Samsung Medical Center, Sungkyunkwan University School of Medicine, 50 Irwon-dong, Gangnam-gu, Seoul 135-710, South Korea

**Keywords:** Heterotaxy syndrome, Single ventricle, Interstage mortality

## Abstract

**Background:**

In spite of improved survival after palliation for single ventricle, interstage mortality for a single ventricle with heterotaxy syndrome is unknown. The purpose of this study was to quantify interstage mortality and influence mortality risk factors.

**Methods:**

From November 1994 until February 2012, all patients that had a functional single ventricle and heterotaxy syndrome who underwent palliative operations at our center were included. Patients with hypoplastic left heart syndrome and operative mortality cases were excluded. The factors that influenced interstage mortality were determined by multivariate Cox analysis.

**Results:**

There were 16 patients with interstage mortality (41.0%), much higher than the non-heterotaxy group (vs. 11.3%, *P* = 0.001, OR = 5.478). The major presumptive causes of death were infection or sepsis (37.5%) and unknown sudden death (31.3%). When we compared the survival group and the mortality group with heterotaxy syndrome, Blalock-Taussig shunt as a 1st palliation is most common for both groups but there were more for the mortality group (81.2% vs. 52.2%), and there were more with bidirectional cavo-pulmonary shunt as a 1st palliation in the survival group (10 patients vs. 2 patients). The existence of pulmonary vein stenosis at initial diagnosis was more common for the mortality group. In multivariate Cox analysis, however, the duration of hospitalization at palliation, the duration of intensive care unit stay after palliation and the existence of pulmonary vein stenosis at diagnosis were significant risk factors.

**Conclusion:**

Interstage mortality for a functional single ventricle with heterotaxy syndrome is significantly higher than for non-heterotaxy syndrome. Therefore more attention should be given to the prevention of interstage mortality in these patients with risk factors.

## Background

Operative survival after a 1st stage operation for Fontan candidates including those with hypoplastic left heart syndrome (HLHS) has significantly improved [1]. This might be due to earlier detection of single ventricular heart disease, improved pre- and post-operative management and refinements in surgical technique. But interstage mortality has been reported to persist at high rates (varying from 10% to 25% for survivors) after a single ventricle palliative cardiac surgery [2-4]. The reasons are not well known but respiratory infection, sepsis, anatomic stenosis, low cardiac output, arrhythmia and sudden cardiac death have been reported as causes for HLHS [1,5]. Improvement of interstage survival using treatments such as a home monitoring program and an active treatment program for feeding difficulties have been used for Fontan candidates, giving significant improvement following a Norwood operation [6]. But there were few studies about interstage mortality after single ventricle palliation in heterotaxy syndrome. Recently Ota et al. reported an improved outcome of surgical management of right isomerism in which the five year survival had improved from 53.8% before 2003 to 81.7% after 2004 [7]. Although there are few reports of improved survival after a modified Fontan operation for heterotaxy syndrome [8,9] we needed to investigate the interstage mortality for these patients. The purpose of this study was to determine the interstage mortality for single ventricle patients with heterotaxy syndrome and compare it to that for non-heterotaxy syndrome. Furthermore, we tried to find risk factors for interstage mortality after a palliative stay for a single ventricle heterotaxy syndrome patient.

## Methods

We conducted a retrospective study of all patients with a functional single ventricle and heterotaxy syndrome who underwent a Fontan operation or died during the interstage period following palliative surgery as a Fontan candidate at Samsung Medical Center from November 1994 until February 2012. This study was approved by the Samsung Seoul Hospital Institutional Review Board and individual consent for the study was waived. Palliative operations included a modified Blalock-Taussig (BT) shunt, pulmonary artery band (PAB) and bidirectional cavopulmonary shunt (BCPS). In this study we excluded patients with a hypoplastic left heart syndrome or a variant and the patients who died early after each palliative operation. The patients who didn’t undergo Fontan operation but were waiting Fontan operation were excluded because 2nd interstage period was not completed. Interstage mortality was defined as mortality that occurred during the period from discharge after the previous palliative operation until before the hospitalization for the next palliative operation. As well, a death that occurred 30 days after the operation was regarded as interstage mortality. Heterotaxy syndrome was classified as right isomerism and left isomerism based on the reviews of echocardiographic and postoperative findings using the criteria proposed by Van Praagh and colleagues [10]. Hypoplastic left heart syndrome or variants were established from echocardiographic findings of obstruction of the systemic outflow and inflow tract that required Norwood operation. The end points for this study were completion of a Fontan operation or death before the next operation as a Fontan candidate. A total of 39 patients were identified for this study. In further analysis we identified 71 single ventricle patients who did not have heterotaxy syndrome or hypoplastic left heart syndrome and operative mortality was excluded.

Patients’ records were carefully reviewed for a diagnosis and each palliative operation. Post-operative deaths were identified from the post-operative medical records. All of the interstage deaths were investigated with the medical records and in an interview by the hospital members. We contact every patient who was lost from our regular follow up and uncovered the death and causes of death. We also gathered information about survival or the cause of death from National Institute of Statistics Korea. For further analysis, some variables for interstage mortality in heterotaxy syndrome were evaluated. We defined hospitalization as an admission for a major palliative cardiac operation and discharge as the last day of hospitalization after a major cardiac operation. We categorized body weight as: under 3rd percentile, from the 3rd to 25th percentile, from the 25th to 50th percentile and more than the 50th percentile considering the normal value for their ages. Valvular regurgitation and pulmonary venous stenosis were checked by a two dimensional echocardiogram by pediatric cardiologists. Pulmonary venous stenosis was observed by echocardiography, cardiac computed tomographic images and simple chest X-ray image. High velocity or continuous pulmonary venous flow in Doppler echocardiography, narrowed diameter of pulmonary venous tract associated with dilated proximal pulmonary vein on tomographic images and pulmonary edema on chest X-ray were defined as pulmonary venous stenosis. And infra-cardiac type of anomalous pulmonary venous return usually regarded as obstructive type.

We compared the cumulative survival rate of the heterotaxy group with that of the non-heterotaxy group by the Kaplan-Meier method and log-rank method. Student’s *t*-test and chi square test were used for a comparison of the two independent groups. Cox analysis was used to analyze the influencing factors of interstage mortality in heterotaxy syndrome. For multivariate analysis, we selected factors that showed a *P* valve less than 0.25 in univariate analysis. The continuous variables are expressed as mean values ± standard deviations. SPSS 19.0 was used for our statistical analysis.

## Results

**Figure 1:**
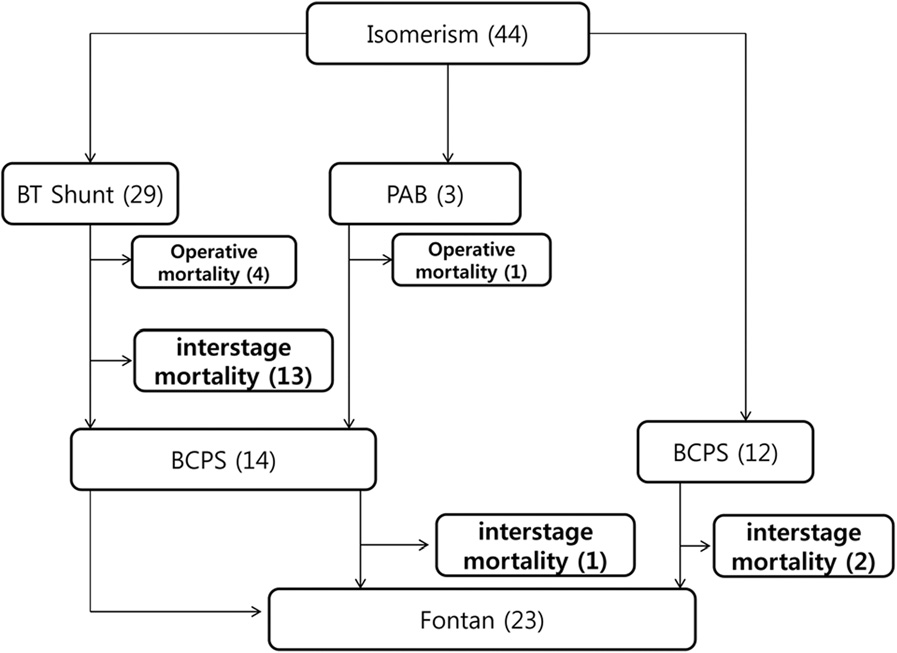
**Among 44 patients of single ventricle with heterotaxy syndrome, except for 5 operative deaths, we experienced 16 interstage deaths.** Therefore 23 patients had completed Fontan procedure.

**Figure 2:**
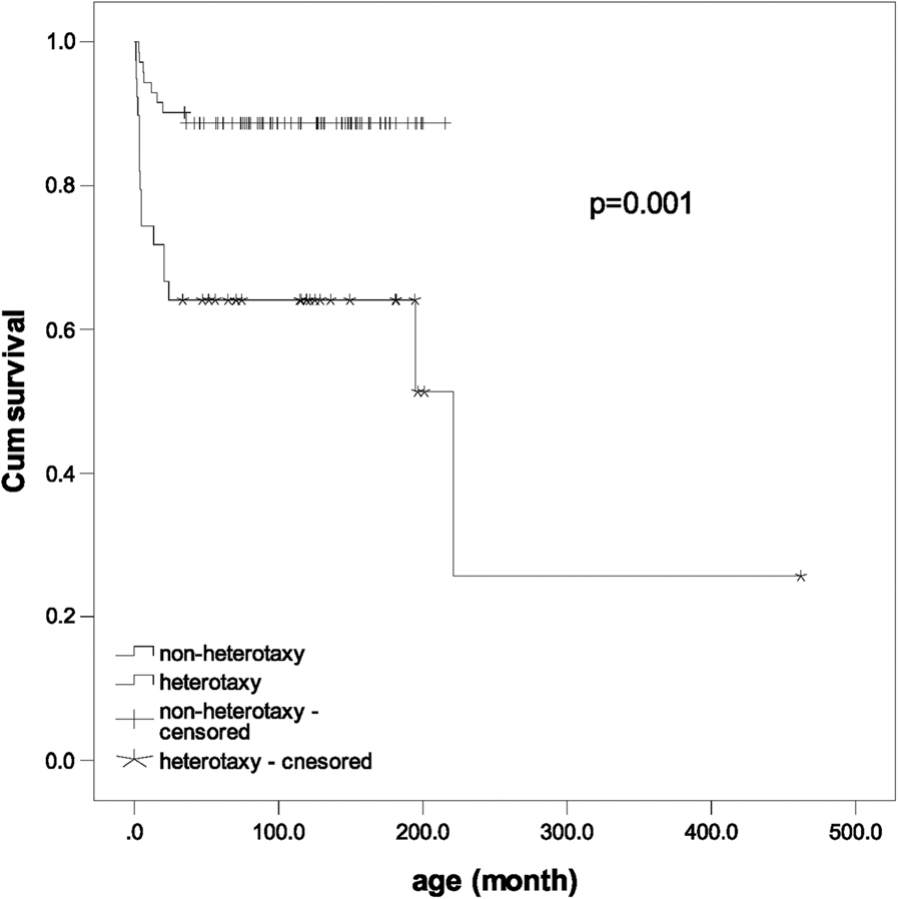
**There is a significant difference in cumulative survival between groups of heterotaxy syndrome and non-heterotaxy syndrome with single ventricle.** The operative mortality cases are excluded in this survival curve.

From 44 patients with functional single ventricle and heterotaxy syndrome who underwent palliative operations, 5 were excluded due to early operative death. Four patients died immediately after the BT shunt palliation and 1 patient died after PAB palliation (Figure 1). Therefore 39 patients were included in this study of which 23 completed their Fontan operation. Of the 39 patients with heterotaxy syndrome, 25 were males (64.1%). There were 33 patients with right isomerism and 4 with left isomerism. The remaining 2 patients had ambiguous morphology. The age at the last follow up was 90.8 ± 93.9 months old. Their first palliations were a modified BT shunt in 25 patients (64.1%), PAB in 2 patients (5.1%) and BCPS in 12 patients (30.8%). The median age at BT shunt or PAB as 1st palliation was 0.8 months of age and the median age of BCPS as 1st palliation was 9.6 months of age. There were 16 patients with interstage mortality (41.0%). On the other hand, 71 patients of the non-heterotaxy group already excluded 9 early operative mortalities (7 patients after BT shunt, 2 patients after BCPS) who had undergone a modified BT shunt (46 patients; 64.8%), PAB (10 patients; 14.1%) and BCPS (15 patients; 21.1%) as a first palliation. The interstage mortality in the non-heterotaxy group was 11.3% (8 patients) with 9.4 months as the median age. There were no significant differences in age, sex, ages of 1st palliation and types of 1st palliation between the heterotaxy syndrome and the non-heterotaxy group. However, the interstage mortality for patients with heterotaxy syndrome was much higher than the non-heterotaxy group (*P* = 0.001) (Table 1) and the cumulative survival rate except for postoperative death was significantly lower for heterotaxy syndrome with a functional single ventricle (*P* = 0.001, OR = 5.4, 95% CI: 2.06-14.50) (Figure 2). The ages of interstage deaths and the time interval from palliation to death were not different significantly between the two groups. There are 9 males in 16 patients of interstage mortality and the median age of death was 4.4 (1.2-221.1) months old and the median time interval from the palliation to death was 2.5 (0.6-62.2) months in the heterotaxy group. The presumptive causes of death were infection or sepsis in 6 patients (37.5%), unknown sudden death in 5 patients (31.3%), arrhythmia in 2 patients (12.5%), pulmonary venous obstruction in 2 patients (12.5%) and heart failure with atrioventricular valve regurgitation in 1 patient (6.3%). We determined sudden death with unknown etiology because some of patients were found to be dead outside our hospital and the others were in state of death on arrival at our hospital. Despite the possible assumptions, we could not confirmed the causes of sudden death due to lack of information. Infection as a cause of death was presumed because laboratory findings on admission showed elevated leukocyte count and inflammatory titer even though no organisms were proven. Some of the patients showed disseminated intravascular coagulation and typical septic feature. Two patients of arrhythmia as a cause of death showed sudden severe bradycardia with atrioventricular block at emergency room and refractory atrial tachycardia in spite of continuing antiarrhythmic agents with sudden ventricular failure at emergency room. Residual stenosis in pulmonary venous inflow was consistently found after operation in two patients who had undergone the corrective operation of pulmonary venous anomaly with obstruction at the first palliation. The 1st palliation was a modified BT shunt in 13 patients (81.3%), PAB in 1 patient (6.3%) and BCPS in 2 patients (12.5%). Thirteen patients with interstage mortality (81.3%) died before BCPS whereas only 3 patients (18.7%) died after BCPS. In the non heterotaxy group, 6 patients with interstage mortality (75.0%) died before BCPS and 2 patients (25.0%) died after BCPS and there was no significant difference from the heterotaxy group (*P* = 0.722).

**Table 1:** Differences between heterotaxy and non-heterotaxy patients

	**Heterotaxy (%)**	**Non-heterotaxy (%)**	***P *value**
**N**	**39**	**71**	
Observation period (mo)	90.8 ± 93.9	107.7 ± 56.1	0.310
Male	25 (64.1)	35 (49.3)	0.163
Major associated anomaly			
Pulmonary atresia	19 (48.7)	23 (32.4)	0.344
Anomalous pulmonary venous return	14 (35.9)	6 (8.5)	0.079
Age of 1st palliation (mo)	3.0 ± 3.1	2.9 ± 6.7	0.907
1st palliation			0.546
BT shunt	25 (64.1)	46 (64.8)	
PAB	2 (5.1)	10 (14.1)	
BCPS	12 (30.8)	15 (21.1)	
Mortality	16 (41.0)	8 (11.3)	**0.001**
Median age of death (range) (mo)	4.4 (1.2-221.1)	9.4 (3.1-35.7)	0.427
Median time to death after palliation (range) (mo)	2.5 (0.6-62.2)	5.1 (1.7-32.3)	0.838
Causes of death			
Infection	6 (37.5)	3 (37.5)	
Sudden death	5 (31.3)	3 (37.5)	
Arrhythmia	2 (12.5)	0	
Pulmonary venous obstruction	2 (12.5)	1 (12.5)	
Heart failure	1 (6.3)	1 (12.5)	
Time of mortality			0.722
Before BCPS	13 (81.3)	6 (75.0)	
After BCPS	3 (18.7)	2 (25.0)	

*BT* Blalock-Taussig, *PAB* pulmonary artery banding, *BCPS* bidirectional cavo-pulmonary shunt.

Table 2 shows the data of the mortality group in functional single ventricle with heterotaxy syndrome compared to those of the survival group in heterotaxy syndrome. The number of patients with right isomerism was more for the mortality group (93.8% vs. 78.3%) and the left isomerism was only for the survival group. In terms of 1st palliation, the BT shunt is the most common palliation for both groups but more in the mortality group (81.2% vs. 52.2%) and the number of patients with BCPS as a 1st palliation was more for the survival group (10 patients vs. 2 patients). The ages at the 1st palliation were significantly higher for the survival group possibly due to a higher portion of BCPS as a 1st palliation (4.1 ± 3.1 vs. 1.5 ± 2.3 months old, *P* = 0.007). It is interesting that body weight at discharge after palliative surgery was lower in the mortality group. Patients with a body weight below the 3rd percentile were 75.0% of total patients while only 17.4% of patients in survival group had a body weight below the 3rd percentile. The existence of pulmonary vein stenosis at initial diagnosis was more common in the mortality group (37.5% vs. 17.4%). The duration of hospitalization, intensive care unit (ICU) stay, and the duration of treatment with mechanical ventilation, intravenous (IV) inotropes and total parenteral nutrition were longer in the mortality group than those of the survival group. The patients who had significant atrial and ventricular arrhythmias took antiarrhythmic agents. Four patients showed atrial tachycardia with frequent premature atrial beats after palliation and one patient showed nonsustained ventricular tachycardia. Amiodarone or sotalol had been used for these patients. There was no significant difference in the medication of antiarrhythmic agents between groups. Oxygen saturation at discharge in the survival group was markedly higher than that of the mortality group in functional single ventricle with heterotaxy syndrome (Table 2).

**Table 2:** The detailed differences between survival group and mortality group in heterotaxy syndrome

	**Survival group (%)**	**Mortality group (%)**
**N**	**23**	**16**
Observation period (mo)	131.1 ± 88.5	32.9 ± 68.9
Male	16 (69.6)	9 (56.3)
Isomerism		
Right	18 (78.3)	15 (93.8)
Left	4 (17.4)	0 (0)
Ambiguous	1 (4.3)	1 (6.3)
Age of 1st palliation (mo)	4.1 ± 3.1	1.5 ± 2.3
1st palliation		
BT shunt	12 (52.2)	13 (81.2)
PAB	1 (4.3)	1 (6.3)
BCPS	10 (43.4)	2 (12.5)
Birth weight (kg)		
< 3p	2 (8.7)	5 (31.3)
3-25p	10 (43.4	6 (37.5)
25-50p	5 (21.7)	3 (18.8)
>50p	6 (26.1)	2 (12.5)
Body weight at discharge (kg)		
< 3p	4 (17.4)	12 (75.0)
3-25p	10 (43.4)	3 (18.8)
25-50p	3 (13.0)	0 (0)
>50p	6 (26.1)	1 (6.3)
AVVR > mild	9 (39.1)	11 (68.8)
Pulmonary atresia	9 (39.1)	7 (43.8)
Pulmonary vein stenosis at diagnosis	4 (17.4)	6 (37.5)
Duration of hospitalization (days)	29.8 ± 17.3	59.1 ± 88.0
Duration of ICU care (days)	9.0 ± 5.9	32.5 ± 48.6
Duration of mechanical ventilation (days)	3.0 ± 2.3	26.4 ± 47.0
Duration of IV inotropics (days)	6.4 ± 6.4	17.8 ± 25.5
Duration of TPN (days)	12.1 ± 11.5	24.0 ± 30.7
Antiarrhythmic at discharge	3 (13.0%)	2 (12.5%)
O2 saturation at discharge (%)	88.1 ± 6.4	78.9 ± 3.3

*BT* Blalock-Taussig, *PAB* pulmonary artery banding, *BCPS* bidirectional cavo-pulmonary shunt, *AVVR* atrioventricular valve regurgitation, *ICU* intensive care unit, *IV* intravenous, *TPN* total parenteral nutrition.

In univariate Cox analysis, the type of 1st palliation when BCPS was compared with BT shunt and PAB, the proportion of low body weight at discharge (< 3 percentile), the duration of treatment with mechanical ventilation and the duration of IV inotropes were significant risk factors for interstage mortality (*P* < 0.05). But in multivariate Cox analysis, the duration of hospitalization at palliation, the duration of ICU stay after palliation and the existence of pulmonary vein stenosis at diagnosis were significant risk factors (Table 3).

**Table 3:** Risk factors for interstage mortality in heterotaxy syndrome with functional single ventricle

	**Univariate Cox analysis (*P *value)**	**Multivariate Cox analysis**		
		***P *value**	**HR**	**95% CI**
Male	0.503			
Isomerism	0.971			
BCPS as the 1st palliation	0.05	0.062	24.4	0.85-699.44
Low Birth weight (<3p)	0.186	0.055	12.7	0.95-170.10
Low Body weight (<3p) at discharge	0.003	0.781	0.7	0.06-7.56
AVVR > mild	0.111	0.373	0.4	0.07-2.68
Pulmonary atresia	0.262			
Pulmonary vein stenosis at diagnosis	0.089	**0.019**	56.5	1.96-1630.41
Duration of hospitalization (days)	0.225	**0.004**	0.8	0.75-0.94
Duration of ICU care (days)	0.055	0.051	1.3	0.99-1.82
Duration of mechanical ventilation (days)	0.040	0.590	0.9	0.78-1.14
Duration of IV inotropics (days)	0.044	0.065	0.8	0.66-1.01
Duration of TPN (days)	0.103	0.116	1.2	0.95-1.55
Antiarrhythmic at discharge	0.590			
Digoxin at discharge	0.617			
Oxygen saturation at discharge (%)	0.002	0.089	0.8	0.66-1.03

*BCPS* bidirectional cavo-pulmonary shunt, *AVVR* atrioventricular valve regurgitation, *ICU* intensive care unit, *IV* intravenous, *TPN* total parenteral nutrition.

## Discussion

It is well known that the prognosis of patients with complex heart disease and heterotaxy syndrome is very poor. Each isomerism has characteristic cardiac anatomical abnormalities. Almost without exception, patients with a right isomerism have obstruction of the pulmonary outflow tract, as well as common mixing situations, and pulmonary atresia is present in two-fifths of cases [11]. More patients with a right isomerism commonly have obstructed anomalous pulmonary venous connection and occasionally they have serious extracardiac anomalies [12]. In contrast to a right isomerism, heart disease in a left isomerism may be relatively mild and a review showed only one-third had complex cyanotic heart disease with a univentricular heart [13]. Interestingly, Asians show a higher prevalence of heterotaxy syndrome compared to Westerners [14] and our series showed 35.5% (39/110) of heterotaxy in Fontan candidates excluded an operative mortality.

We can easily understand that most of our patients were patients with right isomerism and the most of the first palliative procedures were BT shunts. In some cases of well-balanced hemodynamic condition with adequate pulmonary outflow tract obstruction, the first palliation of BCPS is possible. In our study BCPS as a 1st palliation was performed in 30.8% of the total and more in the survival group. It could be the reason that the age of the 1st palliation in survival group was older than that of mortality group. We do not have any special policy about the timing of BCPS with heterotaxy syndrome.

There were various reasons revealed for death. Because much interstage mortality occurred outside of our hospital, it was difficult to find a cause of death for each case. The state of the spleen and the extracardiac anomalies in heterotaxy syndrome may be important risk factors for mortality. In general, a greater frequency of fulminating and fatal septicemia is possible in a right isomerism and that is the reason for the strong recommendation of pneumococcal and H. influenza vaccines. The most frequent cause of interstage death in our patients was infection but we did not have an isolated organism in the six patients who died due to severe infection. Even though infection was also the most important cause of interstage mortality in the non heterotaxy group, infection control and prevention is very important in heterotaxy syndrome with a functional single ventricle in the interstage period.

We had 7 patients who died of unknown sudden causes or arrhythmia (43.8%) among whom only 1 patient had been prescribed antiarrhythmic agents. The causes of interstage mortality in HLHS patients have been extensively studied and it has been described that a preoperative or postoperative history of arrhythmia was associated with a sudden, unexpected interstage mortality [15]. In our study, most of the arrhythmias were detected in ICU care after the palliation. There were no serious arrhythmias that needed to be corrected immediately. We managed significant post-operative arrhythmias medically after immediate post-operative period. In some instances of right isomerism bilateral sinus node activity is present and in left isomerism, true sinus rhythm is less common. In many patients, a progressive slowing of the heart rate has been noted with advanced age, leading to the need for placement of a permanent pacemaker [16,17]. Even though previous arrhythmia history was not a significant risk factor in our study, we were not sure if some of fatal arrhythmia that had not been detected happened to the patients of sudden death.

We also should have an interest in shunt occlusion in patients who underwent BT shunt as 1st palliation. For the prevention of an unexpected sudden interstage death, a comprehensive outpatient program using home pulse-oximetry and daily weight monitoring is useful, but the results were disappointing because interstage mortality did not decrease even with vigilant outpatient monitoring [18]. For the prevention of the thrombosis after shunt operation, aspirin for 6 month after the operation or till the next palliation is usually accepted in our center.

Feeding difficulties after single ventricle palliation are frequently encountered because of a lack of preoperative feeding continued postoperatively, poor oromotor skills or gastroesophageal reflux. In heterotaxy syndrome, gastrointestinal malformation and feeding difficulties are more frequent and associated with aspiration-related death. Interestingly, Hebson et al. reported that neonates undergoing single ventricle palliation who required a gastrostomy and fundoplication were at increased risk of interstage mortality [6]. They suggested feeding difficulty as a marker for an increased risk of interstage mortality. We did not realize the importance of the gastrointestinal malformation and feeding difficulties in heterotaxy syndrome until recently, when frequent vomiting was confirmed from malrotation in one patient. Nowadays we carry out gastrointestinal evaluation in patients with oral feeding difficulties after operation. Unfortunately we cannot provide the complete data of gastrointestinal abnormalities in our series. We suspected aspiration associated with feeding might contribute sudden death in one or two patients. However the interstage mortality in the patients was significantly associated with the body weight at discharge after the major palliative surgery.

Patient growth during the interstage period is a crucial factor and studies have shown that weight gain in infants with HLHS is particularly challenging [19]. The reasons for poor weight gain and low body weight are not clear in heterotaxy syndrome but may be multifactorial. Keller et al. demonstrated a somatic growth delay in patients with HLHS, particularly in the interstage period and also demonstrated that a low weight-for-z score was associated with poorer outcomes including more frequent interstage admission [20]. We did not observed frequent admissions in our patient of interstage death and their feeding status, but the distribution of discharge patient body weight showed that 74.3% of our patients weighed less at discharge, below 25 percentile, while only 58.9% of our patients had their birth weight of below 25 percentile. A special nutritional and feeding program for growth restricted infants in the interstage period was required. With the special program, a higher weight gain allowed them to have next palliative treatment at shorter interval from the 1st palliative treatment [18].

Residual anatomical lesions were not so common in our patients of interstage death but any undetectable minor anatomical problems, especially pulmonary venous obstruction and airway obstruction due to heart failure could contribute to the unexpected sudden death. The types of the 1st palliation were one of three, BT shunt, PAB and BCPS. As for BT shunt size, it depended on the patients’ body weight and surgical approach. Mostly 3.5 mm shunt via sternotomy or 4 mm shunt via lateral thoracotomy was used for our patients. In some cases we reduced the shunt size because of overflow after palliation. When it comes to PAB, external pulmonary artery banding via sternotomy is usual in our center. The strength of bandings is based on Trusler’s formula in single ventricle and is adjusted according to oxygen saturation using hemoclip.

The period before BCPS is accepted as the vulnerable period of sudden cardiac death in HLHS and interstage mortality in the period after the discharge from the first palliation and before BCPS persists at rates varying from 5% to 19% [15]. Therefore from a HLHS study, the authors concluded that early BCPS at less than 4 month of age is safe in spite of a hospital course of longer duration [21]. Our results in heterotaxy syndrome and functional single ventricle showed that the interstage mortality before BCPS was higher than that after BCPS and the cumulative survival of the patients who underwent BCPS as a 1st single ventricle palliation was much better than that of the patients whose 1st palliation were BTS or PAB. Therefore the BCPS as a 1st single ventricle palliation might be superior to BTS or PAB in terms of interstage mortality in heterotaxy syndrome with functional single ventricle and well balanced hemodynamics but further investigation is necessary.

The low oxygen saturation at discharge was one of the risk factors for the interstage mortality in heterotaxy syndrome with palliation for single ventricle. Shunt obstruction has been accepted as a major cause of cyanosis and sudden death in an infant with single ventricle physiology after palliation. Ohman et al. reported that home monitoring of oxygen saturation had the potential to detect some of the life-threatening shunt obstructions after BTS in infants with single-ventricle physiology [22]. But we could not define the level of oxygen saturation of high risk for the interstage home monitoring of oxygen saturation.

Anomalous pulmonary venous drainage with obstruction in a single ventricle and heterotaxy syndrome is still a high risk for operative mortality [23]. In our study, pulmonary venous stenosis at diagnosis was a significant risk factor of interstage mortality by multivariate Cox analysis. Our patients did not show severe pulmonary venous obstructions after the 1st palliation because pulmonary venous anomalies with obstruction are corrected at the initial stage by our policy. The repair of the pulmonary venous connection anomaly did not provoke a higher mortality than nonheterotaxy patients from some reports [5,24], but our results showed that the existence of the pulmonary venous obstruction was an important factor for interstage mortality.

The post-operative course reflected by the duration of hospitalization, ICU stays and IV inotropes after palliation was important for interstage survival in our study. Residual atrioventricular valvular regurgitation was not a risk factor for our patients even though it was reported as a risk factor for late mortality in right isomerism [7]. There was another report that described moderate atrioventricular valvular regurgitation and obstructed pulmonary venous anomaly as risk factors in right isomerism [25]. We tried to correct atrioventricular valvular regurgitation over mild degree as soon as possible and many of correction were performed when BCPS was achieved.

The first limitation of our study is that it is a retrospective study. We tried to identify the mortality cases and the causes of death but we were unable to identify the exact causes of death for some cases that died at other hospitals or at home. We also were unable to identify the presence of a spleen in some patients with heterotaxy syndrome because some medical records were not complete. In general we hypothesized a right isomerism equaled asplenia and recommended preventive vaccination. The vaccination status also failed to be defined for each patient. We excluded a few patients alive that were waiting for BCPS or Fontan operation in a functional single ventricle that made slightly higher mortality rate. There are other important surgical factors that impact on the mortality, such as bypass time, shunt size, strength of banding and aortic regurgitation after palliation. We ignored to analyze some factors because of incompleteness and inaccuracy from old medical record. We focused several risk factors for interstage mortality in heterotaxy syndrome and further extensive investigation will be necessary in this recent period of developed surgical technique.

In summary, the interstage mortality in functional single ventricle and heterotaxy syndrome was much higher than the other non-heterotaxy group. Therefore more attention should be given to the prevention of interstage mortality in these patients.

## Conclusion

Interstage mortality in a functional single ventricle with heterotaxy syndrome was significantly higher than non-heterotaxy syndrome. The duration of hospitalization for palliation, the duration of ICU stay after palliation and the existence of pulmonary vein stenosis at diagnosis were significant risk factors for interstage mortality in heterotaxy syndrome; therefore we need more careful follow-up for the patients with risk factors.

## Abbreviations

HLHS: Hypoplastic left heart syndrome; BT: Blalock-Taussig; PAB: Pulmonary artery band; BCPS: Bidirectional cavopulmonary shunt; AVVR: Atrioventricular valve regurgitation; ICU: Intensive care unit; IV: Intravenous; TPN: Total parenteral nutrition.

## Competing interests

The authors declare that they have no competing interests.

## Authors’ contributions

All authors read and approved the final manuscript. JS, MD: FG, ES, MT; SK, MD: FG; JH, MD: FG; OJL, MD: ES; GK, MD: ES; TGJ, MD: ES; JHY, MD: ES.
